# Cardiovascular Eligibility Criteria and Adverse Event Reporting in Combined Immune Checkpoint and VEGF Inhibitor Trials

**DOI:** 10.1016/j.jaccao.2023.12.010

**Published:** 2024-02-27

**Authors:** Stephen Rankin, Benjamin Elyan, Robert Jones, Balaji Venugopal, Patrick B. Mark, Jennifer S. Lees, Mark C. Petrie, Ninian N. Lang

**Affiliations:** a School of Cardiovascular and Metabolic Health, College of Medical and Veterinary Life Sciences, University of Glasgow, Glasgow, United Kingdom; b NHS Greater Glasgow and Clyde, Glasgow, United Kingdom; c School of Cancer Sciences, College of Medical and Veterinary Life Sciences, University of Glasgow, Glasgow, United Kingdom

**Keywords:** acute coronary syndrome, antiangiogenic therapy, heart failure, hypertension, immunotherapy, ischemia, myocarditis

## Abstract

**Background:**

Combination therapy with immune checkpoint inhibitors (ICIs) and vascular endothelial growth factor inhibitors (VEGFIs) has improved cancer outcomes and is increasingly used. These drug classes are associated with cardiovascular toxicities when used alone, but heterogeneity in trial design and reporting may limit knowledge of toxicities in patients receiving these in combination.

**Objectives:**

The aim of this study was to assess consistency and clarity in definitions and reporting of cardiovascular eligibility criteria, baseline characteristics, and cardiovascular adverse events in ICI and VEGFI combination trials.

**Methods:**

A scoping review was conducted of phase 2 to 4 randomized controlled trials of ICI and VEGFI combination therapy for solid tumors. Trial cardiovascular eligibility criteria and baseline cardiovascular characteristic reporting in trial publications was assessed, and cardiovascular adverse event definitions and reporting criteria were also examined.

**Results:**

Seventeen trials (N = 10,313; published 2018-2022) were included. There were multiple cardiovascular exclusion criteria in 15 trials. No primary trial publication reported baseline cardiovascular characteristics. Thirteen trials excluded patients with prior heart failure, myocardial infarction, hypertension, or stroke. There was heterogeneity in defining cardiovascular conditions. “Grade 1 to 4” cardiovascular adverse events were reported when incidence was ≥5% to 25% in 15 trials. Incident hypertension was recorded in all trials, but other cardiovascular events were not consistently reported. No trial specifically noted the absence of cardiovascular events.

**Conclusions:**

In ICI and VEGFI combination trials, there is heterogeneity in cardiovascular exclusion criteria, reporting of baseline characteristics, and reporting of cardiovascular adverse events. This limits an optimal understanding of the incidence and severity of events relating to these combinations. Better standardization of these elements should be pursued. (Exclusions and Representation of Patients With Kidney Disease and Cardiovascular Disease in Drug Trials of the Novel Systemic Anti-Cancer Therapies VEGF-Signalling Pathway Inhibitors Alone or in Combination With Immune Checkpoint Inhibitors; CRD42022337942)

There is a high prevalence of cardiovascular disease (CVD) among patients with cancer.^1^ The incidence of cardiovascular (CV) events, such as myocardial infarction (MI) and ischemic stroke, is higher in patients with cancer than it is in those without cancer.^2^ As clinical outcomes for people diagnosed with cancer have improved considerably over the past 2 decades, the competing risks from CV comorbidity and mortality have gained increasing relevance.^3^

Therapies such as immune checkpoint inhibitors (ICIs) and vascular endothelial growth factor inhibitors (VEGFIs) have improved cancer outcomes for patients with a variety of tumor types.^3^^,^^4^ When used alone, ICIs are associated with a range of CV adverse events (CVAEs) including myocarditis, MI, and ischemic stroke.^5^^,^^6^ VEGFIs are also associated with a range of CV toxicities, particularly hypertension, as well as left ventricular systolic dysfunction (LVSD), heart failure (HF), and atherothrombotic sequelae including MI and stroke.^7^, ^8^, ^9^

The use of ICIs and VEGFIs in combination is now a common treatment regimen in various cancer types, including melanoma, renal cancer, cervical cancer, and endometrial cancer.^10^ This is a consequence of successful trials of combinations of ICIs and VEGFIs conducted over the past 5 years, with more than 90 ongoing clinical trials of ICI and VEGFI combination regimens.^4^^,^^11^ Six combination ICI and VEGFI treatments are currently approved by the U.S. Food and Drug Administration.^10^ Given the CVAEs seen with each of these drugs in isolation, understanding the potential for an increased incidence of these effects when the drugs are combined is of major importance.

There is limited understanding of the extent to which pre-existing CVD increases the risk for ICI and VEGFI CV toxicity. To understand these issues, it is imperative to have clarity about the representation of patients with or without pre-existing CVD in trials. Understanding and limiting heterogeneity among trial populations is required for subsequent robust meta-analysis of CVAEs. Furthermore, consistency and clarity of definitions and trial publication reporting of CVAEs are fundamental to achieving these aims.

We conducted a scoping review of randomized controlled trials of ICI and VEGFI combination therapy in patients with cancer. Our primary interests were trial CV exclusion criteria and the heterogeneity of these across trials. We also examined reporting of baseline CV characteristics and methods by which adverse events (AEs) were defined, adjudicated, and reported in trial results publications.

## Methods

This scoping review protocol was registered with the International Prospective Register of Systematic Reviews (CRD42022337942) and used the Preferred Reporting Items for Systematic Reviews and Meta-Analyses statement guidance.^12^ We used the population, intervention, comparison, and outcome criteria for inclusion (Supplemental Table 1). The registered protocol also included assessments relating to nephrology-related inclusions and trial reporting, and these findings have been published separately.^13^ As this was a review of publicly available data, no ethics approval was required.

### Search strategy

The search was conducted in MEDLINE, Embase, and the Cochrane Library on May 20, 2022. All trials published in the public domain until the time of data extraction were eligible for analysis. The search terms are included in Supplemental Table 2. Duplicates were removed. Relevant papers were identified by 2 independent reviewers (B.E. and S.R.). Disagreements were resolved by consensus with a third reviewer (J.S.L.).

### Study eligibility criteria

A systematic search of the literature was conducted to identify clinical trials of combination ICI and VEGFI therapy. We included any trial conducted among adult patients with any solid organ cancers who received combination ICI and VEGFI therapy in either the intervention or the control arm. ICIs and VEGFIs that were not approved by the Food and Drug Administration for use as anticancer treatments at the time of data extraction were excluded. Trials using only single dosing or sequential (nonconcurrent) ICI and VEGFI therapy were excluded.

### Inclusion and exclusion criteria

We included all phase 2 to 4 randomized controlled trial with a minimum of 20 participants with available results published at time of extraction. Nonrandomized controlled trials, meta-analyses, review papers, commentaries, subsequent therapy analyses, cost-effectiveness analyses, published abstracts, patient-reported outcomes, subgroup analyses, and retrospective analyses were excluded. If 2 published papers reported data from the same patient group, such as subgroup analyses and extended follow-up analyses, the original publication was used.

### Outcomes

Key trial characteristics, trial eligibility criteria, and exclusion criteria relating to CVD were extracted. Trial design characteristics relating to the assessment and adjudication of CVAEs and the extent of their reporting within the published paper were recorded. Data were extracted from the original publication, supplemental material, and available protocols from the journal website. Trial registration numbers, identified from the publications, were used to search relevant clinical trial platforms to ensure that all relevant publicly available protocol data were identified if they were not available from the publication.

### CVAEs

An AE was defined as a CVAE if it was recorded as a cardiac disorder under the Common Terminology Criteria for Adverse Events (CTCAE) criteria. The CTCAE grade AE severity on a scale of 1 to 5. Grade 1 events are considered “mild” and grade 2 “moderate.” Grade 3 events are considered “severe or medically significant but not immediately life-threatening,” while grade 4 events are those with life-threatening consequences. Death was recorded as grade 5. CVAEs were grouped in similar categories, and of note, MI and “acute coronary syndrome” were reported together under the AE “MI” category. If the AE was not recorded as a cardiac disorder per CTCAE but fulfilled any prespecified trial criteria for CV and stroke endpoints for clinical trials, on the basis of the Food and Drug Administration–endorsed Hicks criteria (such as “sudden death”), it was also classified as a CVAE.^14^

## Results

**Figure 1:**
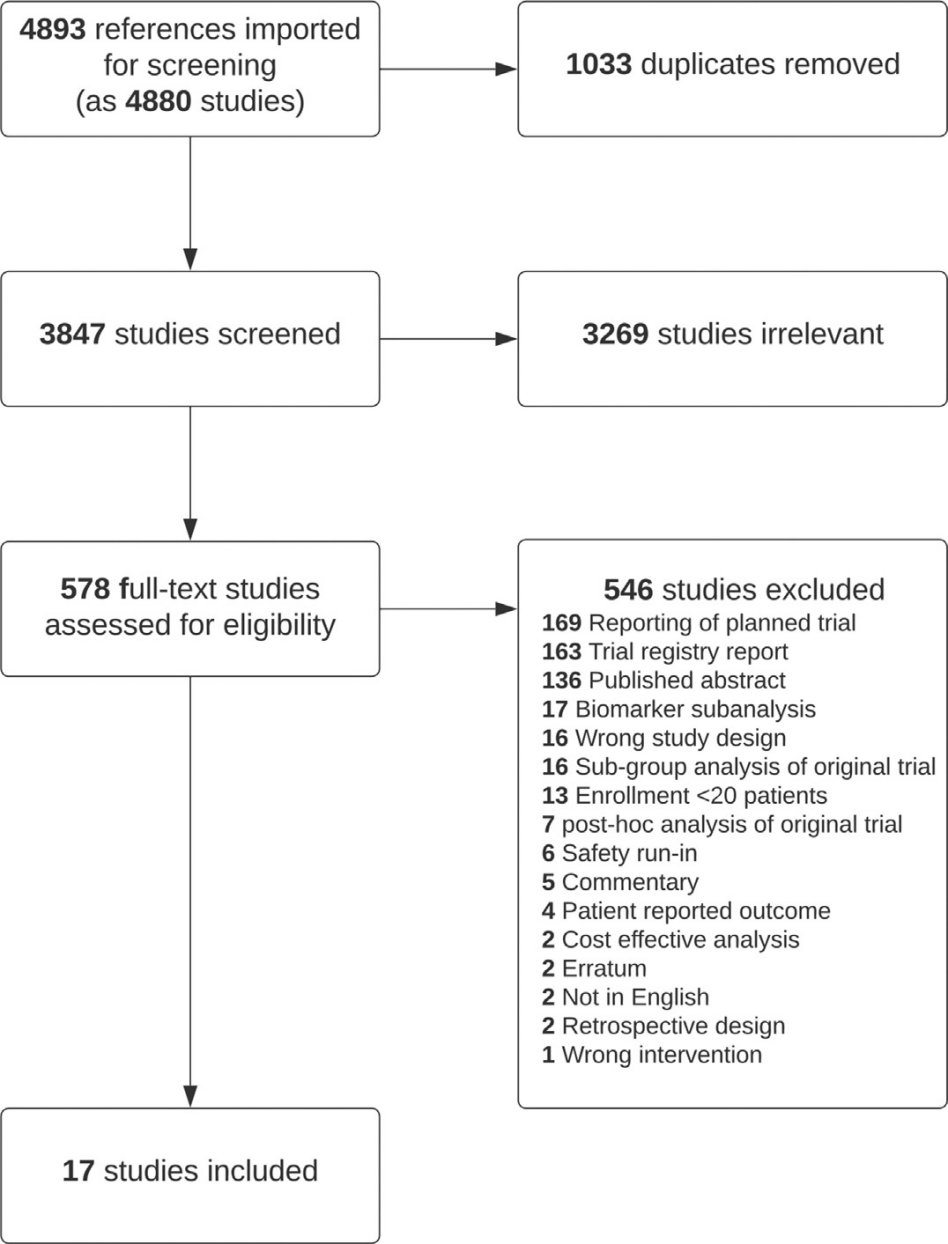
Preferred Reporting Items for Systematic Reviews and Meta-Analyses Diagram Of the 4,893 references extracted, 17 studies were included in the final analysis. Full search terms can be found in Supplemental Table 2.

The search identified 4,893 references, which were screened (Figure 1). The final analysis included 17 randomized controlled trials with a total of 10,313 participants, published between 2018 and 2022 (Table 1).^15^, ^16^, ^17^, ^18^, ^19^, ^20^, ^21^, ^22^, ^23^, ^24^, ^25^, ^26^, ^27^, ^28^, ^29^, ^30^, ^31^, ^32^, ^33^, ^34^, ^35^ Twelve were phase 3 trials (N = 9,687 [94%]) and 5 were phase II (N = 626 [6%]). Eight different combinations of ICIs and VEGFIs were used. Atezolizumab with bevacizumab was the most common combination (Supplemental Table 3), used in 6 trials (N = 4,357 [42%]).

**Table 1:** Randomized Controlled Trials of Immune Checkpoint Inhibitor and Vascular Endothelial Growth Factor Inhibitor Combination Therapy: Exclusion Criteria

First Author (Year)	Combination	N	Tumor Site	NYHA Functional ClassExclusion Criterion	LVEF Exclusion Criterion	Coronary Heart Disease Exclusion Criteria	Blood pressure Exclusion Criterion	Peripheral Arterial Disease Exclusion Criteria	Venous Thromboembolism Exclusion Criteria	Stroke Exclusion Criteria	QTc Interval Exclusion Criteria
Phase 3 RCTs											
André et al (2020)^21^	Pembrolizumab/bevacizumab	307	Bowel	—	—	—	—	—	—	—	—
Choueiri et al (2021)^22^	Nivolumab/cabozantinib	651	Renal	≥IIIb	≤50%	MI, unstable angina, CABG, cardiac angioplasty, or percutaneous coronary intervention^a^	>150/90	Symptomatic peripheral vascular disease	PE/DVT^a^	Stroke/TIA^a^	>450/470
Colombo et al (2021)^23^	Pembrolizumab/bevacizumab	617	Cervical	—	—	—	—	—	—	—	—
Finn et al (2020)^24^	Atezolizumab/bevacizumab	501	Liver	≥IIc	—	MI^b^, unstable angina	≥150/100	Vascular disease^a^^,^^c^	—	Stroke^b^	>500
Makker et al (2022)^25^	Pembrolizumab/lenvatinib	827	Endometrial	≥IIIa	“LLN”	MI, unstable angina^d^	≥150/90	—	—	Stroke^d^	>480
Moore et al (2021)^26^	Atezolizumab/bevacizumab	1,301	Ovarian	≥II	<50%^e^	MI^b^, unstable angina	>150/100	Vascular disease^a^^,^^c^	CTCAE grade 4 VTE	Stroke^b^	—
Motzer et al (2019)^27^	Avelumab/axitinib	886	Renal	Symptomatic^d^	“LLN”	MI, severe/unstable angina, or CABG^d^	≥140/90	Peripheral artery bypass grafting^d^	PE/DVT^a^	stroke/TIA^d^	>500
Motzer et al (2021)^28^	Pembrolizumab/lenvatinib	1,069	Renal	≥IIIa	“LLN”	MI, unstable angina^d^	≥150/90	—	—	Stroke^d^	>480
Rini et al (2019)^29^	Atezolizumab/bevacizumab	915	Renal	≥IIb	<50%	MI, unstable angina^a^	>150/100	Vascular disease^a^^,^^c^	—	Stroke/TIA^a^	>460
Rini et al (2019)^30^	Pembrolizumab/axitinib	861	Renal	≥IIIa	—	MI, unstable angina, CABG, cardiac angioplasty, or stenting^d^	≥150/90	Peripheral artery bypass grafting^d^	PE/DVT^a^	Stroke/TIA^d^	≥480
Socinski et al (2018)^15^	Atezolizumab/bevacizumab	1,202	Lung	≥IIc	<50%^e^	MI, unstable angina^b^	>150/90	Vascular disease^a^^,^^c^	—	Stroke^b^	—
Sugawara et al (2021)^31^	Nivolumab/bevacizumab	550	Lung	≥III	—	MI, unstable angina^a^	≥150/90	—	PE/DVT^a^	Stroke/TIA^a^	—
Phase 2 RCTs											
Lheureux et al (2022)^32^	Nivolumab/cabozantinib	82	Endometrial	≥III	—	MI, unstable angina^a^	>140/90	Thromboembolic event requiring anticoagulation^a^	Thromboembolic event requiring anticoagulation^a^	Stroke/TIA^a^	>500
McDermott et al (2018)^16^	Atezolizumab/bevacizumab	305	Renal	≥II	<50%^e^	MI/unstable angina^b^	>150/100	Vascular disease^a^^,^^c^	—	Stroke/TIA^b^	—
Mettu et al (2022)^33^	Atezolizumab/bevacizumab	133	Bowel	≥II	—	MI, unstable angina, stenting, angioplasty, cardiac surgery,^d^ “active coronary heart disease”	>150/100	Arterial thrombosis,^d^ symptomatic PVD, vascular disease^c^	CTCAE grade 4 VTE	Stroke/TIA^d^	—
Nayak et al (2021)^34^	Pembrolizumab/bevacizumab	80	Brain	∗∗	—	—	Inadequately controlled	Arterial thromboembolism^d^	Thromboembolism^d^	—	—
Redman et al (2022)^35^	Avelumab/bevacizumab	26	Bowel	≥II	—	MI^b^, unstable angina	—	—	—	Stroke^b^	—

CABG = coronary artery bypass graft; CTCAE = Common Terminology Criteria for Adverse Events; DVT = deep vein thrombosis; LLN = lower limit of normal; LVEF = left ventricular ejection fraction; MI = myocardial infarction; PE = pulmonary embolism; QTc = corrected QT; RCT = randomized controlled trial; TIA = transient ischemic attack; VTE = venous thromboembolism.

a Within 6 months.

b Within 3 months.

c Such as aortic aneurysm, dissection or carotid stenosis that requires surgical intervention or stenting, or recent peripheral arterial thrombosis.

d Within 12 months.

e LVEF <50% acceptable if stabilized on optimal medical therapy in the opinion of the treating physician.

### CV eligibility criteria

**Figure 2:**
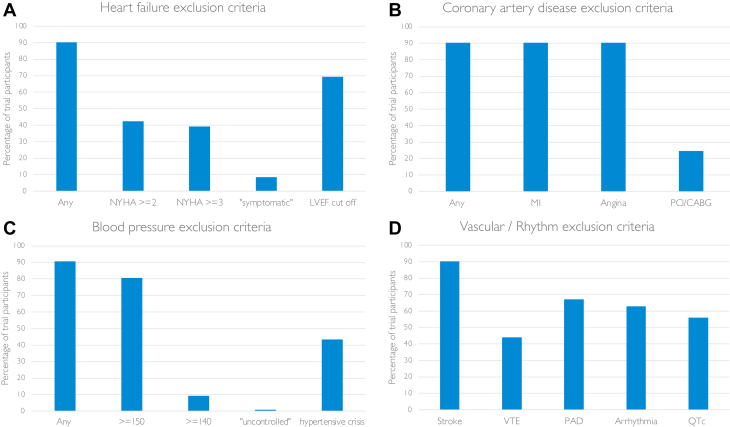
Cardiovascular Exclusion Criteria in Immune Checkpoint Inhibitor and Vascular Endothelial Growth Factor Inhibitor Combination Therapy Trials Percentage of trials with cardiovascular exclusion criteria and the definitions used across trials for (A) heart failure, (B) coronary artery disease, (C) blood pressure, and (D) vascular and rhythm exclusion criteria. CABG = coronary artery bypass graft; LVEF = left ventricular ejection fraction; MI = myocardial infarction; PAD = peripheral artery disease; PCI = percutaneous coronary intervention; QTc = corrected QT interval; VTE = venous thromboembolism.

Eligibility criteria were available for all 17 trials. CVD trial exclusion criteria were broad, with heterogenous definitions (Figure 2). Fifteen trials (N = 9,389 [91%]) had multiple CV exclusion criteria. Of these, there were specific exclusion criteria for patients with prior HF, MI or unstable angina, hypertension, and stroke in 13 trials (N = 9,283 [90%]). Two of the 15 trials (N = 106 [1%]) had a general exclusion criterion of “clinically significant CVD or impairment.” The remaining 2 trials (N = 924 [9%]) did not explicitly exclude patients on the basis of prior CVD but had a general criterion excluding those with “a relevant prior condition that may affect the results of the trial.” The interpretation of these more generic criteria was left to the discretion of the investigator.

In the 12 trials reporting eligibility data prior to enrollment, 31% of participants (n = 3,905) were ineligible. Only 1 paper reported reasons for screening failure, and in that publication, 10% of those ineligible were excluded because of CV exclusions (pulmonary embolism or deep vein thrombosis, hypertension, corrected QT interval, and “CV conditions”).

#### HF and LVSD

Of the 14 trials (N = 9,309 [88%]) with specific exclusions for patients with HF, 7 excluded those with NYHA functional class ≥II, 6 trials excluded those with NYHA functional class ≥III, and 1 trial excluded “symptomatic” patients (Table 1). Eight of the trials’ HF exclusions specified HF within varying time frames prior to enrollment, ranging from 3 to 12 months prior to screening.

Patients with reduced left ventricular ejection fraction (LVEF) were excluded from 8 trials (N = 7,156 [69%]): 5 excluded those with LVEFs <50% (although 3 of these accepted LVEF <50% if the participant was “stable on a medical regimen that was optimized in the opinion of the physician”) and 3 excluded patients with LVEFs less than the “lower limit of normal” of the “institutional normal range.” Only 4 trials (N = 3,433 [33%]) mandated echocardiography before enrollment for all participants. Three other trials (N = 1,909 [19%]) mandated LVEF assessment prior to enrollment in specific circumstances (for patients with anthracycline exposure in 1 trial and if a patient had “cardiac risk factors or abnormal electrocardiographic findings” in the remaining 2 trials).

There were exceptions to allow the inclusion of participants with prior HF. In 4 trials, participants with HF who did not meet prespecified NYHA exclusion criteria, as well as participants with LVEFs <50%, were eligible to enroll provided they were on a stable regimen that was optimized in the opinion of the physician.

#### Coronary artery disease

The 14 trials with LVSD and HF exclusions also excluded patients with histories of recent MI or unstable angina (Table 1). The time frame for exclusion of patients with prior acute coronary syndrome varied from 3 to 12 months prior to screening. In addition to exclusions on the basis of acute coronary syndrome, 4 trials (N = 2,531 [25%]) also excluded patients with coronary angioplasty, stenting, or coronary artery bypass grafting within 6 to 12 months prior to screening. In 4 trials, patients known to have coronary artery disease (not otherwise meeting prespecified coronary exclusions) were eligible for inclusion provided they were on a stable regimen that was optimized in the opinion of the physician.

#### Blood pressure

Fifteen trials had blood pressure or hypertension exclusion criteria (Table 1), most commonly excluding those with systolic blood pressure ≥150 mm Hg (N = 8,315 [81%]). Two trials (N = 106) did not specify blood pressure cutoffs, but 1 trial excluded those with “inadequately controlled hypertension” or histories of hypertensive encephalopathy or crisis. The second trial did not have a specific blood pressure cutoff but excluded participants randomized to receive bevacizumab if they had histories of hypertensive emergency or hypertensive encephalopathy. Any history of hypertensive encephalopathy or crisis was an exclusion criterion in 8 trials (N = 4,463 [43%]).

#### Stroke

Previous “cerebrovascular accident” or transient ischemic attack within 3 to 12 months of screening was an exclusion criterion in 14 trials (N = 9,309 [90%]).

#### Arterial disease

Arterial vascular disease, such as aortic aneurysm requiring surgical repair, peripheral artery bypass grafting, and peripheral arterial thrombosis in the 6 to 12 months prior to screening, was an exclusion criterion in 11 trials (N = 6,917 [67%]). There was heterogeneity in the definition of arterial disease, varying from those with surgical intervention (peripheral artery bypass grafting) to those with any form of intervention or arterial thrombus in the preceding 6 to 12 months. Symptomatic peripheral vascular disease was an exclusion criterion in 2 trials.

#### Venous thromboembolism

“Prior pulmonary embolism or deep vein thrombosis” was an exclusion criterion in 8 trials (N = 4,544 [44%]) (Table 1), 3 of which had time limits of exclusion to within the preceding 6 months. Venous thromboembolism exclusion criteria were defined as either “pulmonary embolism or deep vein thrombosis within the preceding 6 months” or previous “CTCAE grade 4 venous thromboembolism” in 2 trials.

#### Corrected QT interval and arrhythmia

Patients with arrhythmias were excluded from 10 trials (N = 6,482 [63%]). “Unstable” or “hemodynamically significant” arrhythmia was the most common exclusion terminology, but “grade ≥2,” “uncontrolled,” and “clinically significant” arrhythmias were used to define this in 3 trials. Eight trials (N = 5,792 [56%]) had upper limits for corrected QT interval for enrollment, ranging from 450 to 500 ms. Only 1 trial used different thresholds for men and women.

#### Myocarditis

No trial specifically excluded patients with previous myocarditis, but every trial excluded patients with recent or current use of corticosteroids or immunosuppression or previous hypersensitivity to ICI.

### Reporting of baseline CV characteristics

With the exception of smoking status, which was reported in 2 lung cancer trials, no trial reported baseline CV characteristics, such as the prevalence of previous MI, HF, LVSD, diabetes, dyslipidemia, or hypertension.

### Reporting of AEs

All 17 trials reported AEs using CTCAE definitions and severity grading. CTCAE version 4 was used in 15 trials. AEs were reported by the site investigator with no central or CV specialist event adjudication in 14 trials; this was not specified in the remaining 3 trials. One trial had an independent CV event adjudication committee. AEs were reported either as treatment related or “AEs of any attribution.” Treatment-related AEs (adjudicated by the investigator) were reported in all trials. AEs of any attribution were less commonly reported (11 trials, N = 7,458 [72%]).

#### Duration of AE reporting

Follow-up for CV events was shorter than the trial duration in all trials (Table 2). Follow-up for CV events in 5 trials was “the duration of treatment plus 30 days after last dose.” In 9 trials follow-up for CVAEs was “duration of treatment plus 30 days or the initiation of new anticancer therapy, whichever came first.” Ten trials had extended follow-up for serious AEs and AEs of special interest (AEOSIs), including CVAEs, ranging from 90 to 120 days. The follow-up period was not specified in 2 trials.

**Table 2:** Reporting of Common Terminology Criteria for Adverse Events Grade 1 to 4 AEs

First Author (Year)	Combination	Median Safety Follow-Up Duration, mo	Median Efficacy Follow-Up Duration, mo	Threshold Incidence for Reporting AEs in Main Paper	Other Reporting Thresholds for AEs in Paper/Supplement
Andre et al (2020)^21^	Pembrolizumab/bevacizumab	12.1	32.4	≥10%	—
Choueiri et al (2021)^22^	Nivolumab/cabozantinib	17.6	18.1	≥10%	• irAE (all events)
Colombo et al (2021)^23^	Pembrolizumab/bevacizumab	11	22	≥20%	• Comparison of risk difference of AE occurrence between treatment groups with ◦ ≥10% in either arm or ≥5% for grade ≥3 AEs with incidence ≥5% ◦ irAEs (all events)
Finn et al (2020)^24^	Atezolizumab/bevacizumab	9.6	—	≥10%	• Grade 3/4 trAEs ≥2% • Grade 3/4 AEs with incidence 1% • AEs leading to withdrawal ≥1% • AEOSIs (CV): all events
Makker et al (2022)^25^	Pembrolizumab/lenvatinib	8.6	12.2	≥25%	• trAEs ≥10% • AEs leading to dose reduction/interruption ≥5% • AEs leading to discontinuation ≥1% • Clinically significant AEs for lenvatinib (includes CV): all events • AEOSIs for pembrolizumab: all events • SAEs ≥1%
Moore et al (2021)^26^	Atezolizumab/bevacizumab	—	19.9	≥25%, 0.5% for grade ≥3	• SAEs ≥2% • irAEs
Motzer et al (2019)^27^	Avelumab/axitinib	9.6	9.9	≥10%, ≥5% for grade ≥3	—
Motzer et al (2021)^28^	Pembrolizumab/lenvatinib	18	26.6	≥25%	• trAEs ≥10% • A selection of grade ≥3 occurring >10% were reported in main text • CV AEOSIs for ICIs/SACT/VEGFIs: all events
Rini et al (2019)^29^	Atezolizumab/bevacizumab	13	24	≥20%^a^	• trAEs ≥10% • AEOSIs (all events)
Rini et al (2019)^30^	Pembrolizumab/axitinib	11.4	12.8	≥10%	• AEOSIs (all events)
Socinski et al (2018)^15^	Atezolizumab/bevacizumab	7.7	20	≥10%, ≥5% for grade ≥3	• trAE ≥10% or grade 3/4 trAEs ≥1% • trSAEs: all events • Treatment-related irAEs
Sugawara et al (2021)^31^	Nivolumab/bevacizumab	11.5	13.7	≥10%	—
Lheureux et al (2022)^32^	Nivolumab/cabozantinib	—	15.9	≥25%, ≥10% for grade ≥3	“Rare grade 4 trAEs and SAEs” reported
McDermott et al (2018)^16^	Atezolizumab/bevacizumab	10.3	20.7	≥20%^a^^,^^b^	• Single most common AE leading to discontinuation for each drug was reported (proteinuria, AKI, and PPES) • AEOSIs (all events)
Mettu et al (2022)^33^	Atezolizumab/bevacizumab	5.1	20.9	Unspecified^c^	• Treatment related irAEs
Nayak et al (2021)^34^	Pembrolizumab/bevacizumab	—	48.6	≥5% (grade ≥2)	• Grade 4 trAEs: all events
Redman et al (2022)^35^	Avelumab/bevacizumab	—	15.1	All events (grade ≥2)	—

AE = adverse event; AEOSI = adverse event of special interest; AKI = acute kidney injury; CV = cardiovascular; ICI = immune checkpoint inhibitor; irAE = immune-related adverse event; PPES = palmar-plantar erythrodysesthesia syndrome; SACT = systemic anticancer therapy; trAE = treatment-related adverse event; trSAE = treatment-related serious adverse event; VEGFI = vascular endothelial growth factor inhibitor.

a Or AE incidence had a ≥5% difference between arms.

b From supplement: no table in main paper.

c No table: selection of AEs reported in main paper.

#### Incidence thresholds for AE reporting

No phase 3 trial reported all CV events. Fifteen trials reported events when they reached prespecified incidence thresholds (Table 2). The most common threshold reported in the main paper was ≥10% in 6 trials (N = 3,756 [36%]), but higher reporting thresholds (incidence ≥20%-25%) were used in 5 trials (N = 4,307 [42%]). One phase 2 trial (N = 26 [0.25%]) reported all CTCAE grade ≥2 treatment-related AEs. Lower thresholds specifically for reporting more severe AEs (CTCAE grade ≥3 or serious AEs) were used in 9 trials (N = 6,565 [64%]), and those thresholds ranged from “all events” to 10%. Three trials (N = 1,633 [16%]) reported AEs under a variety of other specific circumstances with lower thresholds, such as “AEs leading to discontinuation” (Table 2). Grade 5 AEs (deaths) were reported in all trials. Twelve trials (N = 7,854 [76%]) reported deaths regardless of the relationship to treatment, and 5 trials (N = 2,459 [24%]) reported only treatment-related deaths, adjudicated by the investigators. There was no apparent difference in reporting according to trial phase, sponsorship, or year published (Supplemental Table 4).

### CV events

No trial used the Food and Drug Administration–endorsed, standardized Hicks criteria for reporting of CV events.^14^ With the exception of hypertension, which was reported in all trials, no trial explicitly stated the absence or occurrence of CVAEs. In trial papers that did not report CVAEs other than hypertension, it was not clear whether this was because of a true absence of CVAEs or because of their occurrence with an incidence beneath a reporting threshold.

#### CV death

“AE deaths of any attribution” were reported in 11 trials (N = 7,203), and 7 of these (N = 4,734) reported the mode of death. In 6 trials (N = 3,110), only deaths that were considered to be treatment related (adjudicated by the investigator) were reported.

**Figure 3:**
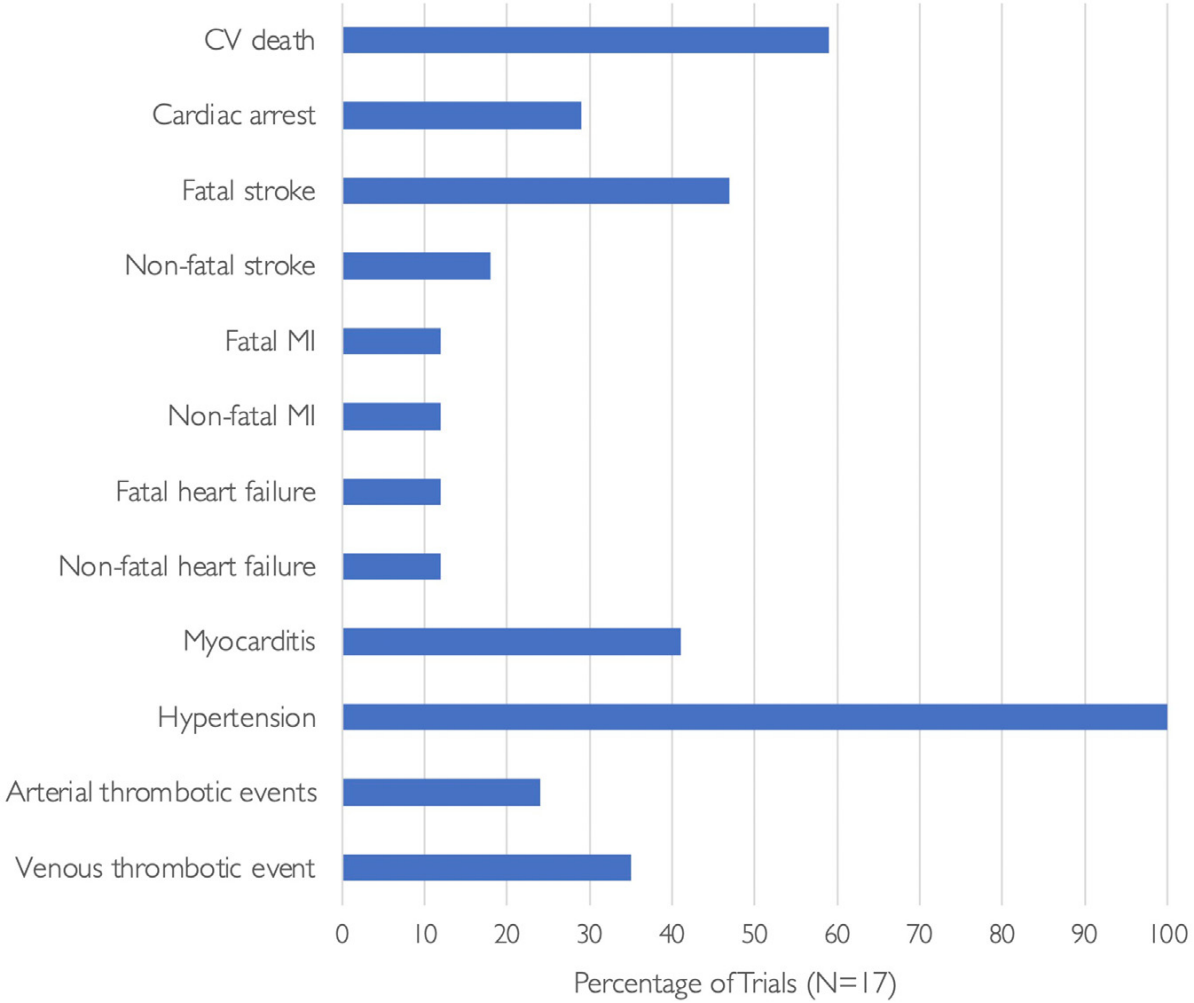
Percentage of Trials Reporting Cardiovascular Adverse Events In ICI and VEGFI combination trials, reporting of cardiovascular (CV) adverse events was variable. With the exception of blood pressure, which was reported in all trials, serious adverse events such as myocardial infarction (MI) were reported in only 4 trials and heart failure in 3 trials. No trial reported the absence of events.

Most frequently, CVAEs were described when associated with death. No trial reported the total number of CV deaths. However, 10 trials (N = 7,737) reported AE deaths that would be categorized as CV deaths according to the Hicks criteria (Figure 3).

### MI

MI was reported in only 4 trials (N = 3,181 [31%]), 2 of which reported only fatal MI (Figure 3). No trial reported whether coronary revascularization occurred.

#### HF and LVSD

HF was reported in 3 trials (N = 2,564 [25%]), 2 of which reported 1 fatal case of HF. One trial reported 1 case of fatal cardiac failure and 3 cases of grade 1 or 2 “congestive cardiac failure” defined according to CTCAE version 4. LVSD was also reported in 3 trials (N = 3,098 [30%]). Two of the 3 trials that reported LVSD mandated echocardiographic surveillance on treatment. Four trials (n = 2,913 [28%]) reported “peripheral edema.”

#### Stroke

Stroke was reported in 8 trials (N = 5,782 [56%]). Five trials (N = 2,778 [27%]) reported only the occurrence of fatal stroke. Ischemic stroke was reported in 5 trials (N = 4,536 [44%]). Fatal ischemic stroke occurred in 4 of these trials, 3 of which were reported in only supplemental data. Hemorrhagic strokes were reported in 6 trials (N = 3,864 [38%]), and 5 of these trials reported only fatal hemorrhagic stroke.

#### Myocarditis

Myocarditis was reported in 7 trials (N = 5,309 [52%]). Fatal myocarditis was reported in 2 trials. No trial reported whether myocarditis did not occur.

#### Hypertension

Hypertension was reported in all trials, defined by the CTCAE. Posterior reversible encephalopathy syndrome was reported in 2 trials (N = 2,271 [22%]). There were 2 reported deaths attributed to hypertension: 1 secondary to posterior reversible encephalopathy syndrome and another secondary to “uncontrolled hypertension” adjudicated by the investigator.

#### Other thrombotic events

Venous thrombotic events were reported in 6 trials (N = 5,309 [52%]), but 4 of these reported only thrombotic events that resulted in death. Four trials (N = 3,599 [35%]) reported arterial thrombotic events, and 3 trials (N = 1,944 [19%]) reported unspecified thromboembolic events.

#### AEOSIs

AEOSIs were collected in 15 trials (N = 10,205 [99%]), all of which included the collection of immune-related AEs, including myocarditis. All 15 trials reported immune-related AEOSIs, with lower incidence thresholds (all immune-related AEOSIs in 13 trials, >1% in the ICI arm in 1 trial, and unspecified in 1 trial).

Additional CV AEOSIs, excluding hypertension and myocarditis, were collected or reported in 6 trials (N = 4,717 [46%]). CVAEs were included in AEOSI lists in the protocols of 5 of these trials (N = 3,890 [38%]). The definition of these CV AEOSIs varied from “grade ≥2 cardiac disorders” to more comprehensive lists detailing reporting of venous thromboembolism, arterial thromboembolism, LVSD, significant arrhythmias, and HF events. Only 4 trials reported CV AEOSIs, but 3 of these reported CV AEOSIs only in supplemental materials (Table 2). No trial specifically reported that AEOSIs did not occur.

## Discussion

This scoping review of randomized trials of ICI and VEGFI combination therapy demonstrates heterogeneity in 3 key areas relevant to potential adverse CV effects of these important anticancer drugs (Central Illustration). First, CV trial exclusion criteria are inconsistent among trials. Second, reporting of the prevalence of CVD and risk factors among trial participants is variable and limited in primary trial papers. Third, there is variation in methods, thresholds, and follow-up periods for reporting and publication of adverse CV events associated with ICI and VEGFI combination therapy.

**Central Illustration:**
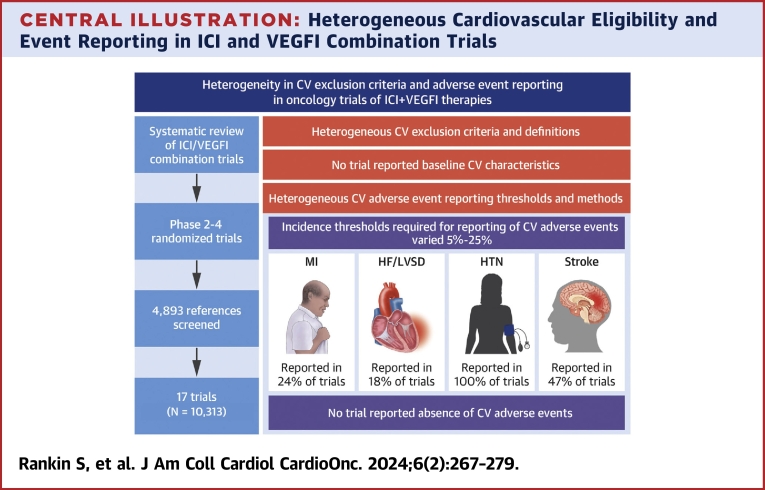
Heterogeneous Cardiovascular Eligibility and Event Reporting in ICI and VEGFI Combination Trials In contemporary trials with “state of the art” trial design, such as trials of combined immune checkpoint inhibitor (ICI) and vascular endothelial growth factor inhibitor (VEGFI) therapy, there is marked heterogeneity in definitions of cardiovascular (CV) disease for exclusion criteria and in adverse event reporting. No trial reported CV baseline characteristics or reported the absence of CV events. CV adverse events were reported only when a threshold incidence within the trial population was reached, which is likely to lead to underreporting of CV events. HF = heart failure; HTN = hypertension; MI = myocardial infarction; LVSD = left ventricular systolic dysfunction.

Randomized trials of combined ICIs and VEGFIs were first reported in 2018 and therefore represent contemporary trial methodology.^15^^,^^16^ A prior review of a broad range of anticancer agents, including conventional chemotherapeutics, examined CVAE reporting in cancer trials supporting Food and Drug Administration approval, but this included trials conducted more than 30 years ago and was prior to any Food and Drug Administration approval of combination therapy.^17^

### CV trial eligibility criteria heterogeneity

Our review identified that CV exclusion criteria were ubiquitous in these trials. We also identified substantial heterogeneity in the nature of these exclusion criteria and the use of potentially arbitrary CV definitions and exclusion thresholds. It is of note that the Food and Drug Administration recommend the avoidance of “unnecessarily restrictive eligibility criteria” to maximize the generalizability of trial results to the patient population in which the drug may be used in subsequent routine clinical practice.^18^ This recommendation was made particularly to allow trials to inform the net risk/benefit profile. Although we acknowledge that it may be appropriate to include some clinically relevant CV eligibility criteria for trial safety reasons, and although these trials were designed and powered to provide information on cancer treatment effects, potential safety signals may become apparent only when trial populations are combined for meta-analysis. Those insights are currently limited by heterogeneity in eligibility criteria.

### Baseline CVD and CVD risk factors in trial participants

The baseline prevalence of CVD, including CV risk factors or established CVD, was not reported in any primary trial publication. However, a secondary analysis of 1 trial did report the prevalence of baseline CV risk factors.^19^ In that trial, the baseline prevalence of CV risk factors was low. Only 4% of patients in the ICI and VEGFI arm had dyslipidemia, 9.5% had diabetes, and 3.2% had cerebrovascular disease.^19^ In addition to potentially stringent trial eligibility criteria, trial recruitment bias toward the inclusion of patients with fewer comorbidities may contribute to a trial population that is not representative of the general population of patients with cancer in whom these drugs may ultimately be used. Irrespective of these issues of eligibility and potential recruitment bias, the lack of data on baseline CV characteristics means that the baseline CV risk for patients in these trials is unknown. Inclusion of those with comorbidities, when assessed in noncancer trials, only modestly affected the completion of study enrollment, meaning that there could be an increase in the generalizability of trial data with minimal impact on trial completion.^20^ Without this information, it is impossible to assess the degree to which pre-existing CVD or risk factors may potentiate adverse CV effects of ICI and VEGFI therapy. It also remains possible that an interaction between pre-existing CVD and adverse effects of ICI and VEGFI therapy is lower than might otherwise be expected. These insights are critical for providing patients with the best information relating to potential risks of treatment in the context of pre-existing CVD.

### CVAE description and reporting

CVAEs were reported using CTCAE in all trials, and their reporting was based upon incidence thresholds. The threshold that was required to be reached varied from 5% to 25% among trials. Furthermore, only 4 trials used lower reporting incidence thresholds for more severe (CTCAE grade ≥3). In addition to the standardization of reporting methods, lowering or potentially removing this threshold for reporting in primary trial publications altogether should be considered. Although the signal-to-noise ratio of grade 1 and 2 events may mean that reporting on the basis of incidence thresholds could be appropriate, we would argue that reporting of all of the more severe AEs may be justified. Irrespective, reporting of events of special interest of any severity should continue for conditions such as myocarditis, for which the most granular information is required to understand whether there may be a potential disconnect between initial CTCAE severity grading and outcomes.

Trial publication reporting of CVAEs, and the clarity of this, was variable. Although many primary trial publications did not report the occurrence of CVAEs, they also did not explicitly state their absence. Reporting of AEs that were specifically considered to have been related to treatment was more frequent than reporting of AEs of any attribution. Reporting of hypertension and, to a lesser extent, myocarditis was common in the context of already well-recognized associations with VEGFIs and ICIs, respectively. However, without consistently robust assessment and reporting of other CVAEs, the ability to discern associations (or the lack thereof) between these drugs and a broader range of potential CVAEs will remain suboptimal. The assessment of CVAE “treatment-relatedness” was by the local investigator, which introduces bias and impedes transparent understanding of AE profiles. One trial included a prespecified subgroup analysis of CV events with ICI and VEGFI therapy. In that analysis, the number of CV events was small, but CVAE incidence was higher than reported in the primary report.^19^

All trials had longer follow-up for anticancer efficacy assessment than they did for the collection of CVAEs. Given that the accrual of CVAEs might be expected to occur over a similarly more prolonged period, increasing follow-up duration for CVAEs would provide important information.

### Study limitations

We did not extract data on pretrial safety data, which may have influenced eligibility criteria. We also did not extract data on subgroup analysis and extended follow-up papers, which may have provided additional information on CV comorbidities and adverse effects. However, given that original trial papers frequently inform drug licensing approvals, we believe that our focus on these publications is particularly relevant. It is also possible that some safety data are still to be placed in the public domain and were therefore not captured. Given that follow-up and trial inclusion time was variable among trials and also among trial participants, reported percentage incidence rates of CVAEs should be considered as crude rates rather than being time adjusted. Data extraction occurred in May 2022. Although further ICI and VEGFI combination trials have been reported since then, we believe that our findings retain relevance, particularly to currently approved combination regimens.

## Conclusions

This scoping review of randomized trials of ICI and VEGFI combination therapies has identified heterogeneity in trial CV eligibility criteria, limited reporting in trial papers of the baseline CV characteristics of participants, and heterogeneity in the methods used to report adverse CV events. These factors may have substantial impact on the ability to make accurate assessments, including meta-analyses, of the potential for CVAEs of these important anticancer therapies. These findings should be considered carefully from the time of inception of novel cancer therapy trials. Our observations have relevance to clinical trialists and to sponsors of research. Importantly, this requires ongoing consideration by regulatory authorities, including the Food and Drug Administration and the European Medicines Agency. Furthermore, alignment and incorporation of consensus definitions of cardiotoxicity, such as those proposed by the International Cardio-Oncology Society, should be considered in the next version of the CTCAE. Although it is possible that CVAEs are underappreciated in cancer trials, it is also possible that they may be less frequent than feared. With the rapid rise of combination ICI and VEGFI treatment regimens, there is an urgent need to standardize these components and, in particular, to inform their use in patients who frequently have pre-existing CVD.

Perspectives **COMPETENCY IN MEDICAL KNOWLEDGE:** ICIs and VEGFIs are now frequently used in combination, often in patients with pre-existing CVD. However, trial data to guide their use in patients with CVD are limited, and there is marked variation in trial exclusion criteria for patients with pre-existing CVD. The representation of patients with pre-existing CVD in these trials is not clear. Without standardized and clear reporting of CV exclusion criteria, baseline characteristics, and event reporting, accurate assessment of CV safety is limited. This has implications when making treatment decisions that require assessment of potential treatment benefits to be weighed against the risk for potential adverse effects.

**TRANSLATIONAL OUTLOOK:** These observations and conclusions from contemporary cancer drug trials have relevance for the design and reporting of the majority of oncology drug trials, irrespective of therapeutic class. There is an opportunity for harmonized trial design and reporting to optimize CV safety assessment. This requires closer collaboration between oncologists and cardiologists.

## Funding Support and Author Disclosures

Drs Petrie and Lang are supported by a British Heart Foundation Centre of Research Excellence Aware (RE/18/6/34217). Dr Rankin has received support through an unrestricted grant from Roche Diagnostics. Dr Lang has received research grants from Roche Diagnostics, AstraZeneca, and Boehringer Ingelheim; and has received consulting and speaker fees from Roche Diagnostics, MyoKardia, Pharmacosmos, Akero Therapeutics, CV6 Therapeutics, Jazz Pharma, and Novartis, all outside the submitted work. Dr Lees has received personal lectureship honoraria from AstraZeneca, Pfizer, and Bristol Myers Squibb, outside the submitted work. Dr Mark has received grants and personal fees from Boehringer Ingelheim; and has received honoraria from AstraZeneca, GlaxoSmithKline, Pharmacosmos, and Astellas, outside the submitted work. Dr Venugopal is a consultant or adviser for Bristol Myers Squibb, EUSA Pharma, and Merck Sharp & Dohme; has received travel and accommodation expenses from Bristol Myers Squibb, EUSA Pharma, and Ipsen; has received research funding to the institution from Bristol Myers Squibb, Exelixis, Ipsen, Merck Sharp & Dohme, and Pfizer; has received honoraria from Bristol Myers Squibb, Ipsen, and Pfizer; and is a Speakers Bureau member and has provided expert testimony for Bristol Myers Squibb, Eisai, EUSA Pharma, Merck Serono, Merck Sharp & Dohme, and Pfizer. Dr Jones has received grants from Astellas, Clovis, Exelixis, Bayer, and Roche; has received honoraria from Astellas, Janssen, Bayer, Pfizer, Merck Serono, Merck Sharpe & Dohme, Novartis, Roche, Ipsen, and Bristol Myers Squib. Dr Petrie has received grants from Boehringer Ingelheim, Roche, SQ Innovations, AstraZeneca, Novartis, Novo Nordisk, Medtronic, Boston Scientific, Horizon, and Phramacosmos, all outside the submitted work; has received honoraria from Boehringer Ingelheim, Novartis, AstraZeneca, Novo Nordisk, AbbVie Bayer, Takeda, Corvia, Cardiorentis, Pharmacosmos, Siemens, and Vifor. Dr Elyan has reported that he has no relationships relevant to the contents of this paper to disclose.

## References

[1] Battisti N.M.L., Welch C.A., Sweeting M. (2022). Prevalence of cardiovascular disease in patients with potentially curable malignancies. J Am Coll Cardiol CardioOnc.

[2] Paterson D.I., Wiebe N., Cheung W.Y. (2022). Incident cardiovascular disease among adults with cancer. J Am Coll Cardiol CardioOnc.

[3] Topalian S.L., Hodi F.S., Brahmer J.R. (2019). Five-year survival and correlates among patients with advanced melanoma, renal cell carcinoma, or non–small cell lung cancer treated with nivolumab. JAMA Oncol.

[4] Heo J.H., Park C., Ghosh S., Park S., Zivkovic M., Rascati K.L. (2021). A network meta-analysis of efficacy and safety of first-line and second-line therapies for the management of metastatic renal cell carcinoma. J Clin Pharm Ther.

[5] Drobni Z.D., Alvi R.M., Taron J. (2020). Association between immune checkpoint inhibitors with cardiovascular events and atherosclerotic plaque. Circulation.

[6] Johnson D.B., Balko J.M., Compton M.L. (2016). Fulminant myocarditis with combination immune checkpoint blockade. N Engl J Med.

[7] de Wit S., Glen C., de Boer R.A., Lang N.N. (2022). Mechanisms shared between cancer, heart failure, and targeted anti-cancer therapies. Cardiovasc Res.

[8] Chen D.-Y., Liu J.-R., Tseng C.-N. (2022). Major adverse cardiovascular events in patients with renal cell carcinoma treated with targeted therapies. J Am Coll Cardiol CardioOnc.

[9] Dobbin S.J.H., Mangion K., Berry C. (2020). Cardiotoxicity and myocardial hypoperfusion associated with anti-vascular endothelial growth factor therapies: prospective cardiac magnetic resonance imaging in patients with cancer. Eur J Heart Fail.

[10] U.S. Food and Drug Administration. Drugs@FDA: FDA approved drugs. https://www.accessdata.fda.gov/scripts/cder/daf/index.cfm.

[11] Huinen Z.R., Huijbers E.J.M., van Beijnum J.R., Nowak-Sliwinska P., Griffioen A.W. (2021). Anti-angiogenic agents—overcoming tumour endothelial cell anergy and improving immunotherapy outcomes. Nat Rev Clin Oncol.

[12] National Institute for Health Research Centre for Reviews and Dissemination, University of York International Prospective Register of Systematic Reviews. https://www.crd.york.ac.uk/prospero/.

[13] Elyan B.M.P., Rankin S., Jones R., Lang N.N., Mark P.B., Lees J.S. (2023). Kidney disease patient representation in trials of combination therapy with VEGF-signaling pathway inhibitors and immune checkpoint inhibitors: a systematic review. Kidney Med.

[14] Hicks K.A., Mahaffey K.W., Mehran R. (2018). 2017 cardiovascular and stroke endpoint definitions for clinical trials. Circulation.

[15] Socinski M.A., Jotte R.M., Cappuzzo F. (2018). Atezolizumab for first-line treatment of metastatic nonsquamous NSCLC. N Engl J Med.

[16] McDermott D.F., Huseni M.A., Atkins M.B. (2018). Clinical activity and molecular correlates of response to atezolizumab alone or in combination with bevacizumab versus sunitinib in renal cell carcinoma. Nat Med.

[17] Bonsu J.M., Guha A., Charles L. (2020). Reporting of cardiovascular events in clinical trials supporting FDA approval of contemporary cancer therapies. J Am Coll Cardiol.

[18] U.S. Food and Drug Administration Cancer Clinical trial eligibility criteria: patients with organ dysfunction or prior or concurrent malignancies: guidance for industry. https://www.fda.gov/media/123745/.

[19] Rini B.I., Moslehi J.J., Bonaca M. (2022). Prospective cardiovascular surveillance of immune checkpoint inhibitor–based combination therapy in patients with advanced renal cell cancer: data from the phase III JAVELIN Renal 101 trial. J Clin Oncol.

[20] Hanlon P., Butterly E., Shah A.S. (2022). Assessing trial representativeness using serious adverse events: an observational analysis using aggregate and individual-level data from clinical trials and routine healthcare data. BMC Med.

[21] André T., Shiu K.-K., Kim T.W., Jensen B.V. (2020). Pembrolizumab in microsatellite-instability–high advanced colorectal cancer. N Engl J Med.

[22] Choueiri T.K., Powles T., Burotto M. (2021). Nivolumab plus cabozantinib versus sunitinib for advanced renal-cell carcinoma. N Engl J Med.

[23] Colombo N., Dubot C., Lorusso D. (2021). Pembrolizumab for persistent, recurrent, or metastatic cervical cancer. N Engl J Med.

[24] Finn R.S., Qin S., Ikeda M. (2020). Atezolizumab plus bevacizumab in unresectable hepatocellular carcinoma. N Engl J Med.

[25] Makker V., Colombo N., Casado Herráez A. (2022). Lenvatinib plus pembrolizumab for advanced endometrial cancer. N Engl J Med.

[26] Moore K.N., Bookman M., Sehouli J. (2021). Atezolizumab, bevacizumab, and chemotherapy for newly diagnosed stage III or IV ovarian cancer: placebo-controlled randomized phase III trial (IMagyn050/GOG 3015/ENGOT-OV39). J Clin Oncol.

[27] Motzer R.J., Penkov K., Haanen J. (2019). Avelumab plus axitinib versus sunitinib for advanced renal-cell carcinoma. N Engl J Med.

[28] Motzer R., Alekseev B., Rha S.-Y. (2021). Lenvatinib plus pembrolizumab or everolimus for advanced renal cell carcinoma. N Engl J Med.

[29] Rini B.I., Powles T., Atkins M.B. (2019). Atezolizumab plus bevacizumab versus sunitinib in patients with previously untreated metastatic renal cell carcinoma (IMmotion151): a multicentre, open-label, phase 3, randomised controlled trial. Lancet.

[30] Rini B.I., Plimack E.R., Stus V. (2019). Pembrolizumab plus axitinib versus sunitinib for advanced renal-cell carcinoma. N Engl J Med.

[31] Sugawara S., Lee J.-S., Kang J.-H. (2021). Nivolumab with carboplatin, paclitaxel, and bevacizumab for first-line treatment of advanced nonsquamous non-small-cell lung cancer. Ann Oncol.

[32] Lheureux S., Matei D.E., Konstantinopoulos P.A. (2022). Translational randomized phase II trial of cabozantinib in combination with nivolumab in advanced, recurrent, or metastatic endometrial cancer. J Immunother Cancer.

[33] Mettu N.B., Ou F.-S., Zemla T.J. (2022). Assessment of capecitabine and bevacizumab with or without atezolizumab for the treatment of refractory metastatic colorectal cancer. JAMA Netw Open.

[34] Nayak L., Molinaro A.M., Peters K. (2021). Randomized phase II and biomarker study of pembrolizumab plus bevacizumab versus pembrolizumab alone for patients with recurrent glioblastoma. Clin Cancer Res.

[35] Redman J.M., Tsai Y.-T., Weinberg B.A. (2022). A randomized phase II trial of mFOLFOX6 + bevacizumab alone or with AdCEA Vaccine + avelumab immunotherapy for untreated metastatic colorectal cancer. Oncologist.

