# Supplementary material for: Cardiovascular Eligibility Criteria and Adverse Event Reporting in Combined Immune Checkpoint and VEGF Inhibitor Trials
Source: JACC CardioOncol. 2024 Feb 27;6(2):267–79. doi: 10.1016/j.jaccao.2023.12.010 (PMC11103039; doi:10.1016/j.jaccao.2023.12.010)
Supplement: Supplementary Material [file mmc1.docx]

**Supplemental Table 1: PICO framework table.**

### Outcomes of interest using PICO framework and data points of interest

| Population | Inclusion criteria:  - adult population with cancer,  - all solid organ tumour sites (including lymphoma).  Exclusion criteria:  - animal studies. |
| --- | --- |
| Intervention | Inclusion criteria:  - trials that included the use of a VEGF-inhibitors (VEGFI), and/or in combination with immune checkpoint inhibitors (ICIs) class of drugs for the treatment of cancer,  - VEGFI with or without ICI used as either control arm or intervention arm of study,  - phase II, III and IV trials, - randomised studies, - published in English language,  - completed enrollment,  The specific intervention exclusion criteria are listed below:  - single dosing,  - sequential therapy rather than concurrent therapy, - population of treatment group of <20 patients,  -non-randomised trials  - meta-analysis,  - review articles or commentaries, - subsequent therapy analysis,  - cost-effective analysis,  - published abstracts,  - patient reported outcomes,  - subgroup analysis,  - duplications,  - retrospective analysis.  Trials that have incomplete protocol and text will be included in the results as part of our objective is to identify the representation of patients with kidney disease in trial data. |
| Comparison | Not applicable |
| Outcome | We will collect the following data points from the extracted articles regarding the exclusion and representation of patients with cardiovascular disease:  1. Were patients excluded with cardiovascular disease?  2. Were patients excluded with the following diagnosis:  - Hypertension  - Coronary artery disease (CAD)  - Heart failure  - Cardiomyopathy  - Arrhythmia  - Valvular disease  - Thromboembolic disease  - Cerebrovascular disease  - Abnormal ECG  - Myocarditis  - Pericarditis  - Vasculitis  3. Are the diagnosis of cardiovascular disease of trial population available in baseline characteristics or supplementary materials?  4. Are cardiovascular adverse events reported in the published clinical trial or supplement?  We will also collect the following information about the trial characteristics to analysis if this had an influence on the trial design or patient enrolment:  1. Is the trial ID number identifiable?  2. Is the trial name identifiable?  3. What is the trial design?  4. Was this a randomised control design?  5. Was this intervention or control?  6. Was there an active comparator?  7. Trial year published  8. Trial population size  9. Funding source  10. Cancer diagnosis  11. Is trial protocol available?  12. How were adverse events defined and were they adjudicated?  13. Is there a clear safety follow up period specified in the protocol?  14. Is data collected on adverse events of special interest?  15. Are adverse events of special interest defined in the protocol? |

**Supplemental Table 2: List of VEGF inhibitors and immune checkpoint inhibitors & systematic review search terms**

| **VEGF inhibitors** | |
| --- | --- |
| **Tyrosine kinase Inhibitor** | |
| Apatinib  Axitinib  Brivanib alaninate  Cabozantinib  Cediranib  Dovitinib  Lenvatinib  Nintedanib | Pazopanib  Regorafenib  Sorafenib  Sunitinib  Tivozinib  Vandetanib  Vatalanib |
| **Monoclonal antibodies** | |
| Bevacizumab | Ramucirumab |
| **VEGF-Trap mediators** | |
| Aflibercept | |
| **Immune checkpoint inhibitors** | |
| **PD-1** | |
| Pembrolizumab  Nivolumab | Cemiplimab |
| **PD-L1** | |
| Atezolizumab  Avelumab | Durvalumab |
| **CTLA-4** | |
| Ipilimumab | Tremelimumab |
| **Search Terms** | |
| ((“Phase II” OR “Phase 2” OR "Phase III" OR "Phase 3" OR "Phase IV" OR "Phase 4") AND  (Ipilimumab OR Tremelimumab OR Atezolizumab OR Avelumab OR Durvalumab OR Pembrolizumab OR Nivolumab OR Cemiplimab) AND  (Apatinib OR Axitinib OR Bevacizumab OR Aflibercept OR 'Brivanib alaninate' OR Cabozantinib OR Cediranib OR Dovitinib OR Ramucirumab OR Lenvatinib OR Nintedanib OR Pazopanib OR Regorafenib OR Sorafenib OR Sunitinib OR Tivozanib OR Vandetanib OR Vatalanib)) | |
| Abbreviations: VEGF, Vascular endothelial growth factor; PD-1, Programmed cell death protein-1; PD-L1, Programmed death ligand-1; CTLA-4, Cytotoxic T-lymphocyte protein-4 | |

**Supplemental Table 3. Summary of trial characteristics and exclusions**

|  | **Number of trials n (%)** | **Number of patients n (%)** |
| --- | --- | --- |
| Total (randomized controlled trials) | 17 | 10313 |
| Median Age* | 62 (50-67) | |
| Male | 5308 (51%) | |
| Female | 5005 (49%) | |
| Overall Median safety follow-up* | 11 (5.1-18.0) | |
| Overall Median efficacy follow up* | 19.9 (9.9 – 48.6) | |
| **Tumour type** | | |
| Renal | 6 (35) | 4687 (45) |
| Gynaecological (endometrial, ovarian, cervical) | 4 (24) | 2827 (27) |
| Lung | 2 (12) | 1752 (17) |
| Liver | 1 (6) | 501 (5) |
| Bowel | 3 (18) | 466 (5) |
| Brain | 1 (6) | 80 (1) |
| **Trial Phase** | | |
| III | 12 (71) | 9687 (94) |
| II | 5 (29) | 626 (6) |
| **ICI and VEGFi combination** | | |
| Atezolizumab & bevacizumab | 6 (35) | 4357 (42) |
| Pembrolizumab & Lenvatinib | 2 (12) | 1896 (18) |
| Pembrolizumab & bevacizumab | 3 (18) | 1004 (10) |
| Avelumab & axitinib | 1 (6) | 886 (9) |
| Pembrolizumab & axitinib | 1 (6) | 861 (8) |
| Nivolumab & cabozantinib | 2 (12) | 733 (7) |
| Nivolumab & bevacizumab | 1 (6) | 550 (5) |
| Avelumab & bevacizumab | 1 (6) | 26 (0.3) |
| *weighted by trial participants | | |

**Supplemental Table 4. CVAE reporting by trial phase, sponsorship and year published.**

| **CV event** | **Number of trials (number of participants)** | **Trial phase** | | **Trial Sponsorship** | | **Year trial published** | |
| --- | --- | --- | --- | --- | --- | --- | --- |
|  |  | **II** | **III** | **Industry** | **Academic** | **2018-June 2020** | **July 2020-2022** |
| Total | 17 (10313) | 5 (626) | 12 (9687) | 9 (7239) | 8 (3074) | 7 (4977) | 10 (5336) |
| CV death | 10 (7737) | 2 (385) | 8 (7352) | 8 (6588) | 2 (1149) | 6 (4670) | 4 (3067) |
| Cardiac arrest | 5 (3963) | 0 | 5 (3963) | 4 (2894) | 1 (1069) | 3 (2277) | 2 (1686) |
| Fatal Stroke | 8 (5782) | 2 (385) | 6 (5397) | 7 (5702) | 1 (80) | 5 (3784) | 3 (1998) |
| Non-fatal Stroke | 3 (3004) | 0 | 3 (3004) | 3 (3004) | 0 | 2 (1703) | 1 (1301) |
| Fatal MI | 2 (1478) | 0 | 2 (1478) | 2 (1478) | 0 | 1 (861) | 1 (617) |
| Non-fatal MI | 2 (1703) | 0 | 2 (1703) | 2 (1703) | 0 | 2 (1703) | 0 |
| Fatal HF | 2 (2063) | 0 | 2 (2063) | 2 (2063) | 0 | 2 (2063) | 0 |
| Non-fatal HF | 2 (1703) | 0 | 2 (1703) | 2 (1703) | 0 | 2 (1703) | 0 |
| Myocarditis | 7 (5384) | 1 (133) | 6 (5251) | 3 (3148) | 4 (2336) | 3 (2054) | 4 (3330) |
| Hypertension | 17 (10313) | 5 (626) | 12 (9697) | 9 (7239) | 8 (3074) | 7 (4977) | 10 (5336) |
| Arterial thrombotic events | 4 (3599) | 0 | 4 (3599) | 2 (1703) | 2 (1896) | 2 (1703) | 2 (1896) |
| Venous thrombotic events | 6 (5309) | 0 | 6 (5309) | 5 (4482) | 1 (827) | 3 (2564) | 3 (2745) |
